# A Novel Bioactive Peptide, T14, Selectively Activates mTORC1 Signalling: Therapeutic Implications for Neurodegeneration and Other Rapamycin-Sensitive Applications

**DOI:** 10.3390/ijms24129961

**Published:** 2023-06-09

**Authors:** Sanskar Ranglani, Anna Ashton, Kashif Mahfooz, Joanna Komorowska, Alexandru Graur, Nadine Kabbani, Sara Garcia-Rates, Susan Greenfield

**Affiliations:** 1 Neuro Bio Ltd., Building F5, Culham Science Centre, Abingdon OX14 3DB, UK; anna.ashton@neuro-bio.com (A.A.); kashif.mahfooz@neuro-bio.com (K.M.); joanna.komorowska@neuro-bio.com (J.K.); sara.garciarates@neuro-bio.com (S.G.-R.); susan.greenfield@neuro-bio.com (S.G.); 2 School of Systems Biology, George Mason University, Fairfax, VA 22030, USA; agraur@gmu.edu (A.G.); nkabbani@gmu.edu (N.K.)

**Keywords:** mTORC1, Alzheimer’s disease, cancer, T14, rapamycin, NBP14, T30, acetylcholinesterase

## Abstract

T14 modulates calcium influx via the α-7 nicotinic acetylcholine receptor to regulate cell growth. Inappropriate triggering of this process has been implicated in Alzheimer’s disease (AD) and cancer, whereas T14 blockade has proven therapeutic potential in in vitro, ex vivo and in vivo models of these pathologies. Mammalian target of rapamycin complex 1 (mTORC1) is critical for growth, however its hyperactivation is implicated in AD and cancer. T14 is a product of the longer 30mer-T30. Recent work shows that T30 drives neurite growth in the human SH-SY5Y cell line via the mTOR pathway. Here, we demonstrate that T30 induces an increase in mTORC1 in PC12 cells, and ex vivo rat brain slices containing substantia nigra, but not mTORC2. The increase in mTORC1 by T30 in PC12 cells is attenuated by its blocker, NBP14. Moreover, in post-mortem human midbrain, T14 levels correlate significantly with mTORC1. Silencing mTORC1 reverses the effects of T30 on PC12 cells measured via AChE release in undifferentiated PC12 cells, whilst silencing mTORC2 does not. This suggests that T14 acts selectively via mTORC1. T14 blockade offers a preferable alternative to currently available blockers of mTOR as it would enable selective blockade of mTORC1, thereby reducing side effects associated with generalised mTOR blockade.

## 1. Introduction

T14, a 14mer peptide cleaved from the enzyme acetylcholinesterase (AChE) [1], has a trophic role in early development [2,3]. However, its aberrant activation later in adulthood is thought to drive the pathology of Alzheimer’s disease (AD) [2,3,4]. T14 enhances calcium influx via an allosteric site at the α-7 nicotinic acetylcholine receptors (α-7 nAChR) [3,5], promoting cell growth via a previously unidentified process [3]. However, its inappropriate activation in the context of a mature system can lead to functionally excitotoxic levels of calcium influx [2,4,5,6], resulting in neurodegeneration [2,4]. The levels of T14 increase in the human brain as the pathology of AD progresses [7,8]. The blockade of T14 action by a cyclic version of the peptide, NBP14 [7], has shown therapeutic potential, attenuating neurodegeneration in vitro [9], ex vivo [10], and in vivo [8]. NBP14 acts by displacing its linear counterpart on the α-7 nAChR [11], thereby blocking its downstream effects, which has been validated in post-mortem human brain [8]. Moreover, T14 has also been implicated in driving metastasis [12,13], and its blockade by NBP14 suppresses the migration of cancer cells [13]. Given the association of T14 with both cancer and AD [13], we aimed to explore further the possible common mechanism underlying these two otherwise very different disorders [14].

The mammalian target of rapamycin (mTOR) is a nutrient and energy sensor [15,16], and controls protein synthesis and growth [17,18,19]. When activated, it exists in two forms, mTOR complex 1 [20], and mTOR complex 2 (mTORC1 and mTORC2, respectively) [21]. Hyperactivation of mTORC1 has been implicated in driving the pathology of AD [22], and cancer [23,24]. The presence of mTORC1 in the brain increases as AD pathology advances [22,25], suggesting that the corresponding increasing levels of T14 may be interlinked. T14 is cleaved from the C-terminus of AChE [1], which has several trypsin like cleavage points, thereby allowing for the production of a 30mer peptide, T30 [7,9], with similar bioactive properties to T14 [7,9]. A recent study showed that T30 exposure within human SH-SY5Y cells increased mTOR pathway activation during neurite growth [26]. Both T14 and mTORC1 are involved in growth as trophic agents [3,18,19,26], however, their inappropriate activation is suggested to have an instrumental role in driving similar pathologies [1,2,22,24]. We wished to explore if this correlation could actually be causally linked i.e., whether T14 could act as an extracellular signalling molecule which drives the activation of the intracellular mTORC1 pathway via calcium signalling at the α-7 nAChR, leading to its downstream effects.

Rapamycin is a blocker of the mTOR pathway [27], and its action on the mTOR has been characterised previously [28,29]. However, rapamycin and other mTOR blockers entail a large number of side effects [27,30,31,32]. In a retrospective cohort study of kidney transplant patients, it was reported that 98% of patients administered the drug experienced side effects and this resulted in a high discontinuation rate of 46% [30]. Thus, there is an unmet need to identify a treatment which can target mTORC1 hyperactivation that nonetheless offers the prospects of fewer side effects [33]. We explore here whether T14 blockade could be used as comparable therapy for mTORC1 hyperactivation.

We have previously shown that the application of T30 leads to an increased release of AChE by PC12 cells in a dose-dependent manner [9], which is blocked by NBP14 [7]. Moreover, in the ex vivo rat brain slices, application of T30 results in an increase in the expression of amyloid beta and T14 [11], whereas blockade using NBP14 brings the levels of these neurodegenerative markers down to control levels [11].

Here we show that the levels of T14 and phosphorylated mTOR at serine residue 2448 (p-mTOR s2448, marker for active mTORC1 [34,35]) correlate in midbrain samples from patients of AD. We subsequently demonstrate that T30 drives an increase in the expression of p-mTOR s2448 both in PC12 cells and in brain slices containing substantia nigra of the ex vivo rat brain; in contrast T30 has no effect on phosphorylated mTOR at serine residue 2481 (marker for active mTORC2, [34,35]). This demonstrates that T30 selectively activates mTORC1. We then determine whether the intracellular signalling downstream of T30 is mediated by mTOR. Indeed, the blockade of the mTOR complex using rapamycin attenuates the T30-induced increase in AChE release in differentiated PC12 cells. However, since long term administration of rapamycin (24 h in our study) is known to have off-target effects of both the mTOR complexes [36], mTORC1 and mTORC2, we assess via which specific pathway T30 acts. Accordingly, we silence *Raptor* (required for the formation of the mTORC1 [25]) or *Rictor* (required for the formation of mTORC2 [37]) and observe the impact of these genetic manipulations on AChE release induced by T30. Silencing *Raptor* attenuates T30 induced AChE release by PC12 cells; however silencing *Rictor* does not. Furthermore, the T30-driven increase in p-mTOR s2448 in PC12 cells is blocked by NBP14, however there is no effect on p-mTOR s2481, suggesting that NBP14 can selectively block mTORC1 activation by T30. Blockade of T14 may provide an alternative strategy for treating mTORC1 hyperactivation, with fewer side effects.

## 2. Results

### 2.1. T14 and p-mTOR s2448 Correlate in Midbrain Samples from Alzheimer’s Disease Patients

**Figure 1:**
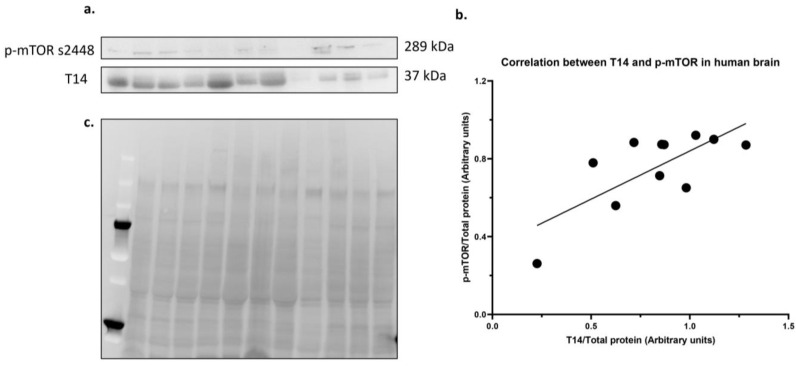
Correlation between T14 and p-mTOR in human midbrain samples from AD patients. (**a**) Western blot image. p-mTOR s2448 (289 kDa) and T14 (37 kDa). (**b**) Scatterplot showing the log transformed values of T14 (*x*-axis) and p-mTOR s2448 (*y*-axis), normalised to the total protein stain (arbitrary units). (**c**) Total protein stain for the Western blot used as loading control. *n* = 11.

We investigated the possible correlation between T14 and p-mTOR s2448 levels by performing a Western blot in midbrain samples from Alzheimer’s disease patients (Figure 1a). Based on a Pearson’s correlation test, there was a significant positive correlation between T14 and p-mTOR s2448 (Figure 1b, *p* = 0.0104, two-tailed) with a strong correlation coefficient, r = 0.7325. Total protein stain was used to normalize for the amount of protein loaded in the Western blot (Figure 1c).

### 2.2. T30 Increases the Expression of p-mTOR s2448 in Differentiated PC12 Cells and Ex Vivo Rat Brain Slices Containing Substantia Nigra

**Figure 2:**
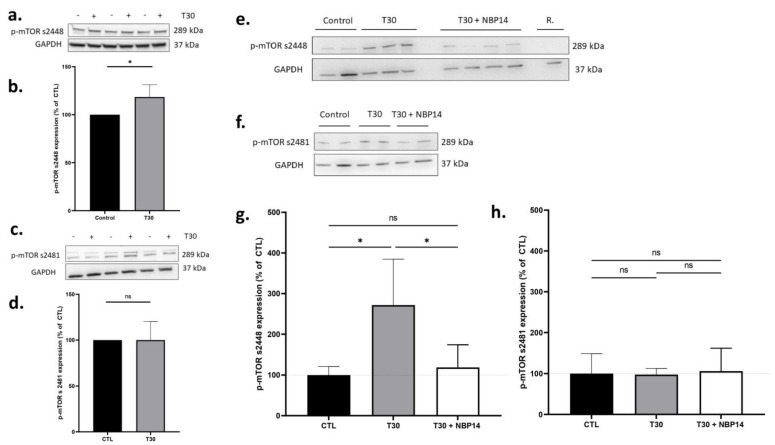
T30 induces an increase in the expression of p-mTOR s2448, but not p-mTOR s2481, in PC12 cells and ex vivo brain slices containing substantia nigra. (**a**) Representative Western blot for T30 (2 µM) treated rat brain slices containing the substantia nigra region for p-mTOR s2448 (289 kDa) and GAPDH (37 kDa). (**b**) Average expression of p-mTOR s2448 in T30 (2 µM) vs. vehicle control treated rat brain slices containing the substantia nigra region (normalised to GAPDH). (**c**) Representative Western blot for T30 (2 µM) treated rat brain slices containing the substantia nigra region for p-mTOR s2481 (289 kDa) and GAPDH (37 kDa). (**d**) Average expression of p-mTOR s2481 in T30 (2 µM) vs. vehicle control treated rat brain slices containing the substantia nigra region (normalised to GAPDH). (**e**) Representative Western blot for T30 (100 µM), T30 (100 µM) + NBP14 (10 µM), R. (rapamycin, 1 µM) or vehicle control treated PC12 cells for p-mTOR s2448 (289 kDa) and GAPDH (37 kDa). (**f**) Representative Western blot for T30 (100 µM), T30 (100 µM) + NBP14 (10 µM), or vehicle control treated PC12 cells for p-mTOR s2481 (289 kDa) and GAPDH (37 kDa). (**g**) Average expression of p-mTOR s2448 in T30 (100 µM), T30 (100 µM) + NBP14 (10 µM) or vehicle control treated PC12 cells (normalised to GAPDH). (**h**) Average expression of p-mTOR s2481 in T30 (100 µM), T30 (100 µM) + NBP14 (10 µM) or vehicle control treated PC12 cells (normalised to GAPDH). The bars represent the mean and the errors represent the SD. *n* = 3–7. * *p* < 0.05. ns: non-significant based on a one-way ANOVA followed by a Dunnett’s post-hoc correction.

We aimed to determine if T30 application increased the expression of p-mTOR protein in differentiated PC12 cells and ex vivo rat brain slices, in the substantia nigra region. Accordingly, T30 was introduced into the cell culture media for 6 h in vitro, or the ex vivo aCSF in which the brain slices were incubated for 5 h. T30 induced a significant increase in the expression of p-mTOR s2448 in ex vivo rat brain slices containing substantia nigra, at 118% of the control (Figure 2a,b, one sample *t*-test, *p* = 0.0171), however T30 had no effect on p-mTOR s2481 expression (Figure 2c,d, one sample *t*-test, *p* = 0.9998). Similarly, in PC12 cells, T30 induced a significant increase in p-mTOR s2448 compared to control (271% of control, Figure 2e,g), which was blocked by NBP14 (118% of control, one-way ANOVA, F = 6.835, *p* = 0.0104, R^2^ = 0.5325, Figure 2e,g). However, both T30 alone and T30 with NBP14 had no effect on p-mTOR s2481 expression in PC12 cells (one-way ANOVA, F = 0.0406, *p* = 0.9607, R^2^ = 0.0088, Figure 2f,h). Rapamycin-treated PC12 cells were included as a negative control: this completely abolished the expression of p-mTOR s2448 as expected (Figure 2e).

### 2.3. Rapamycin Attenuates the T30 Induced Increase in AChE Release by Differentiated PC12 Cells

**Figure 3:**
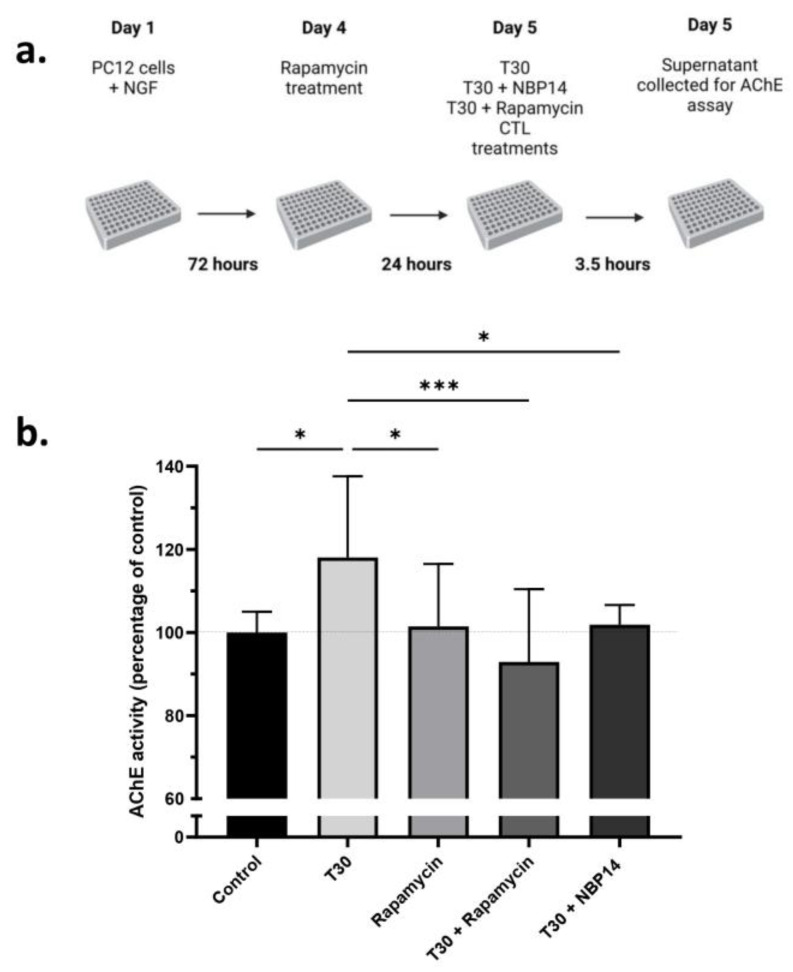
Rapamycin blocks T30 induced increase in AChE release in differentiated PC12 cells. (**a**) Experiment outline. Cells were plated with NGF 72 h before rapamycin treatment. The cells were pre-treated with 1 µM rapamycin for 24 h before the treatments were added. The treatments used were as follows: T30 (40 µM), rapamycin (1 µM), NBP14 (10 µM), or vehicle control (CTL). (**b**) AChE activity measured in cell supernatants. The bars represent the mean, and the error bars represent the SD. One-way ANOVA followed by a Dunnett’s correction to compare the mean of each condition to T30. *n* = 10–12, * *p* < 0.05, *** *p* < 0.001.

T30 induced a significant increase in AChE release by differentiated PC12 cells (one way ANOVA; F = 5.159, *p* = 0.0014, R^2^ = 0.2881) which was blocked by rapamycin and NBP14 (Figure 3). A post-hoc test followed by Dunnett’s correction revealed that rapamycin significantly attenuated the T30 induced increase in AChE release (*p* = 0.0002), which was comparable to the effect of NBP14 (*p* = 0.0337). Rapamycin alone had no effect on the release of AChE release by differentiated PC12 cells (significantly different than T30, *p* = 0.0288).

### 2.4. Silencing Raptor, but Not Rictor Blocked T30 Induced Increase in AChE Release by Undifferentiated PC12 Cells

**Figure 4:**
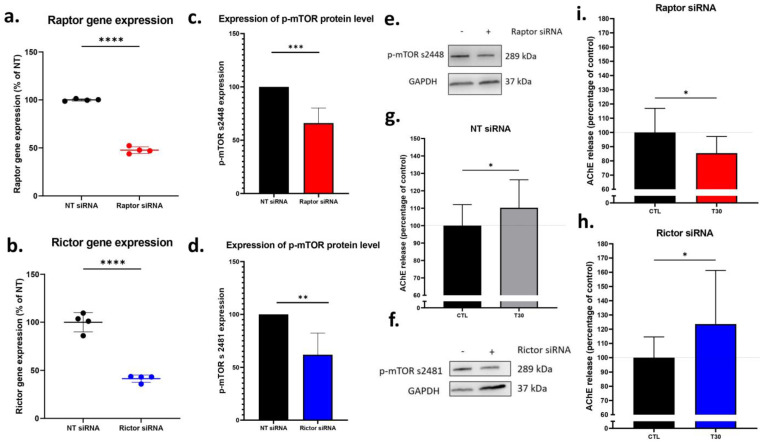
Silencing raptor, but not rictor, reverses the T30 induced increase in AChE release by undifferentiated PC12 cells. (**a**) Average gene expression of raptor as determined by RT-qPCR as a percentage of the NT-siRNA group (*n* = 4). (**c**) Average protein expression of p-mTOR s2448 in raptor silenced cell lysates (*n* = 8). (**e**) Representative Western blot exhibiting downregulation of p-mTOR s2448 in raptor silenced cell lysates, compared to non-targeting siRNA treated cell lysates. (**b**) Average gene expression of rictor as determined by RT-qPCR as a percentage of the NT-siRNA group (*n* = 4). (**d**) Average protein expression of p-mTOR s2481 in rictor silenced cell lysates (*n* = 8). (**f**) Representative Western blot exhibiting downregulation of p-mTOR s2481 in rictor silenced cell lysates, compared to non-targeting siRNA treated cell lysates. (**g**) T30 (20 µM) induced an increase in AChE release by NT-siRNA treated cells (grey bar) shown as a percentage of control (*n* = 19–20). (**h**) T30 (20 µM) induced an increase in AChE release by rictor silenced cells (blue bar) as a percentage of control (*n* = 14). (**i**) T30 (20 µM) induced a decrease in AChE release by raptor silenced cells (red bar) as a percentage of control (*n* = 14). The bars represent the mean, and the error bars represent the SD. A *t*-test was performed to assess the significance between the means of two groups. * *p* < 0.05. ** *p* < 0.01. *** *p* < 0.005. **** *p* < 0.0001.

**Figure A1:**
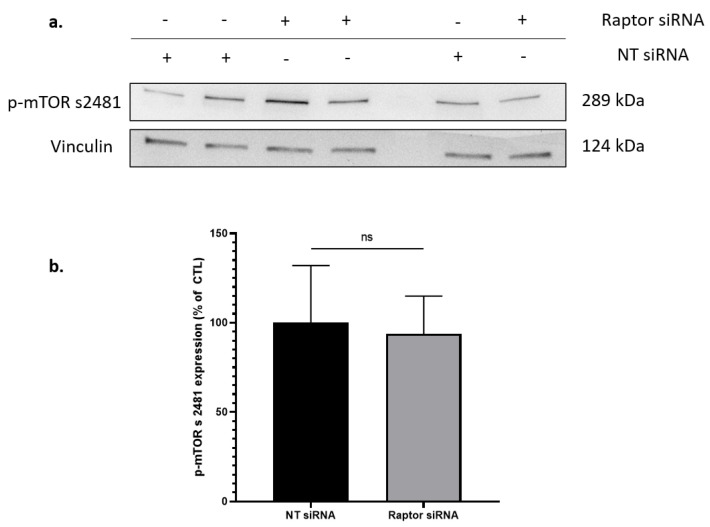
Silencing Raptor has no off-target effects on p-mTOR s2481. (**a**) Western blot image. p-mTOR s2481 (289 kDa) normalised to vinculin loading control (124 kDa). (**b**) Average expression of p-mTOR s2481 in non-targeting siRNA (NT-siRNA) vs. raptor siRNA treated PC12 cells represented as a percentage of control. The errors represent the SEM. ns: non-significant based on a *t*-test.

**Figure A2:**
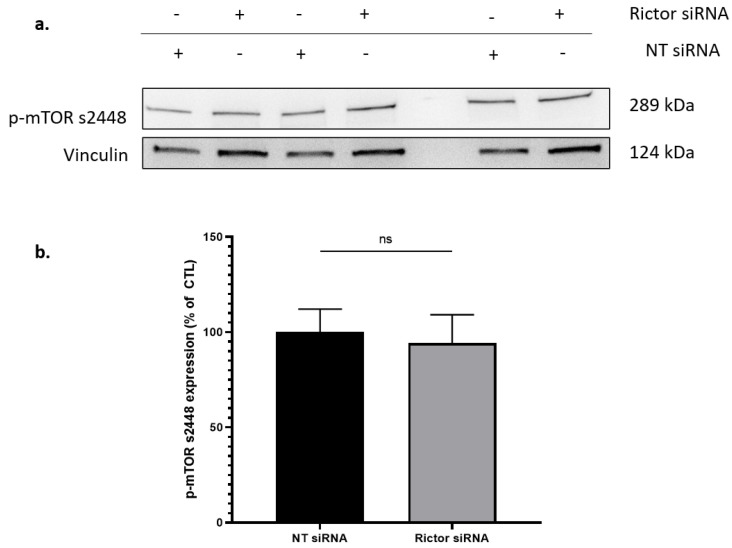
Silencing rictor has no off-target effects on p-mTOR s2448. (**a**) Western blot image. p-mTOR s2448 (289 kDa) normalised to vinculin loading control (124 kDa). (**b**) Average expression of p-mTOR s2448 in non-targeting siRNA (NT-siRNA) vs. rictor siRNA treated PC12 cells represented as a percentage of control. The errors represent the SEM. ns: non-significant based on a *t*-test.

In the next study, we scrutinized the potential mTOR complexes which are targeted by T30 application. The mTORC1 pathway requires raptor (regulatory-associated protein of mTOR), whereas the mTORC2 pathway requires rictor (rapamycin-insensitive companion of mTOR) for their activation, whilst abolishing them blocks the formation of these complexes. Accordingly, we silenced *Raptor* to inactivate the mTORC1 pathway, and *Rictor* to inactivate the mTORC2 pathway. RT-qPCR analysis demonstrated successful siRNA silencing; *Raptor* mRNA expression in the raptor siRNA group was significantly lower than the non-targeting (NT) siRNA group (Figure 4a, unpaired *t*-test, *p* < 0.0001). Similarly, *Rictor* mRNA expression in the rictor siRNA group was significantly lower than the NT siRNA group (Figure 4b, unpaired *t*-test, *p* < 0.0001). Western blotting was then performed to confirm that there was a subsequent inactivation of the respective mTOR complexes following siRNA silencing of *Rictor* and *Raptor*. Antibodies against p-mTOR phosphorylated at different residues (s2448 for mTORC1 and s2481 for mTORC2) were used because it has been previously shown that silencing either *Raptor* or *Rictor* differentially affects the phosphorylation of mTOR [35]. Western blot confirmed that upon *Raptor* silencing, the expression of p-mTOR s2448 was downregulated (Figure 4c,e, one sample *t*-test, *p* = 0.0003). Whilst *Rictor* silencing reduced the expression of p-mTOR s2481 (Figure 4d,f, one sample *t*-test, *p* = 0.0012). Silencing either *Rictor* or *Raptor* had no effect on the p-mTOR s2448 or p-mTORs2481, respectively, confirming that silencing was limited to their respective mTOR complexes with no off-target effects (see Figure A1 and Figure A2). We then assessed whether abolishing mTORC1 or mTORC2 influences the downstream effects of T30 application in PC12 cells by measuring the release of AChE. In the NT siRNA group, T30 induced a significant increase in AChE release in PC12 cells as expected (Figure 4g, unpaired *t*-test, *p* = 0.0308). A similar effect was observed in the *Rictor* silenced group, with T30 inducing an increase in AChE release (Figure 4h, unpaired *t*-test, *p* = 0.0458). However, in the *Raptor* silenced group, T30 induced a decrease in AChE release (Figure 4i, unpaired *t*-test, *p* = 0.01350).

### 2.5. Rapamycin Reduces the Number of Viable Undifferentiated PC12 Cells

**Figure 5:**
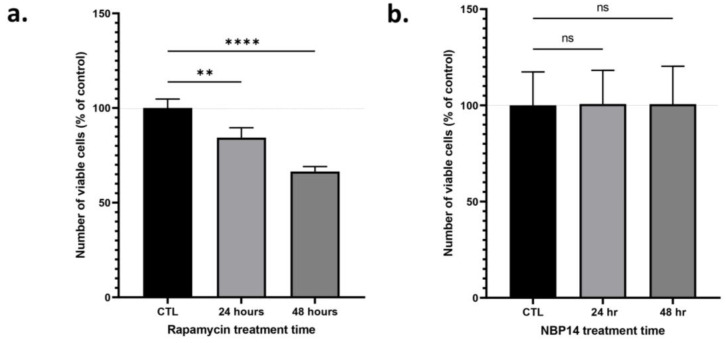
Rapamycin reduces the number of viable PC12 cells, but NBP14 does not. Cells were treated with either (**a**) Rapamycin (1 µM) or (**b**) NBP14 (1 µM) or vehicle control for 24 or 48 h before their viability was determined. The bars represent the mean number of viable cells as a percentage of their respective control. The error bars represent the SD. One-way ANOVA followed by a Dunnett’s post-hoc test. ** *p* < 0.01. **** *p* < 0.0001. ns: non-significant.

Rapamycin (1 µM) reduced the number of viable undifferentiated PC12 cells, as determined by a cell viability assay (Figure 5a, one-way ANOVA, F = 60.01, *p* < 0.0001, R^2^ = 0.9302). A post-hoc test followed by Dunnett’s correction revealed that rapamycin significantly reduced the viability of PC12 cells at 24 h (*p* = 0.0012), and 48 h (*p* < 0.0001). However, NBP14 (1 µM) did not affect the viability of PC12 cells (Figure 5b, one-way ANOVA, F = 0.01304, *p* = 0.9871, R^2^ = 0.0002).

## 3. Discussion

### 3.1. In-Vitro, Ex Vivo and Post-Mortem Preparations

PC12 cells are produced from a pheochromocytoma cell line derived from the rat adrenal medulla [38], are readily cultured, and can be differentiated to display neuron-like behaviour by the addition of NGF [39]. They are derived from the neural crest [40], and release several different neurotransmitters [41], thereby offering a ‘window to the brain’ by providing a useful tool to study neuronal function and dysfunction in vitro, including neurodegeneration [39,41,42]. The adrenal medulla of AD patients also exhibits several pathological features [43], which are comparable to those seen in the brain [7]. Moreover, exogenous T14/T30 has similar effects on PC12 cells comparable to amyloid beta across a range of parameters [6,7]. Both amyloid beta and T14 induce a dose-dependent increase in calcium influx, which is excitotoxic and leads to decreased cell viability, in turn resulting in release of compensatory AChE by the extant cells [6,7,9]. However, the parameter of T30- evoked calcium influx was deemed inappropriate for this current study as it is dependent on the α-7 nAChR on the membrane [9], thus the blockade or manipulation of the mTOR pathway inside the cells would be too upstream for it to be impacted. Similarly, the parameter of cell viability would not be feasible, since the pharmacological blockade of the mTOR pathway itself affects the proliferation rate of PC12 cells [44]. Hence, for the current study, the only remaining parameter which could be used to study the effect of mTOR manipulation was compensatory AChE release. Both T14 and T30 treatments lead to a dose-dependent increase in the release of AChE by undifferentiated PC12 cells, which is blocked by NBP14, by displacing its linear counterpart on the α-7 nAChR [6,7,9]. Here, we blocked the mTOR pathway and tested if T30 could still induce an increase in AChE release in PC12 cells.

The ex vivo rat brain preparation is a novel method to study the impact of bioactive agents such as T30 in a physiological scenario [11,45]. This approach allows for the measurement of responses to T30 in different regions of the brain for the medium term (up to 5 h) [11]. Previous studies using this methodology were performed in the basal forebrain region of post-natal day 14 rat brain [11]; however, in the current study the substantia nigra of the post-natal day 21 rat was investigated. The substantia nigra is abundant in α-7 nAChR [10], the receptor via which T30 acts [3,26]. It is also a part of the ‘isodendritic core’ [46], the group of nuclei derived from the basal plate of the embryo [47], which retain their developmental potential into adulthood and are first to degenerate during the onset of AD [48]. Optical imaging studies demonstrate that T30 has a similar effect on evoked neuronal responses measured by voltage sensitive dye imaging (VSDI) in the substantia nigra region of the P21 rat brain as it does in the P14 basal forebrain, which is blocked by NBP14 [10]. Therefore, use of the rat brain slices containing substantia nigra of P21 rats was considered an appropriate ex vivo model to study the effects of T30 in a more physiological preparation than tissue cultures.

Finally, midbrain samples from AD patients were used to determine the correlation between p-mTOR s2448 and T14. While p-mTOR is increased in the hippocampus of AD patients [25], we chose the midbrain since it would be directly comparable to the ex vivo studies (above) and also since T14 levels increase in the midbrain in a BRAAK stage dependent manner [7,8]. Moreover, the midbrain of AD patients is primarily vulnerable to degeneration before the pathology of AD spreads to the cortex and hippocampus, and before the cognitive symptoms of dementia manifest [46,47,48]. Therefore, midbrain was chosen as the region of interest to assess the correlation between T14 and p-mTOR. Western blotting was considered appropriate to study the amount of these proteins present in the brain: this technique allows the measurement of these proteins in the same samples, as we could stain for both the proteins of interest on the same membrane, thus eliminating any cross-experimental inconsistencies. The polyclonal T14 antibody is specific to the full sequence of T14 and does not recognize AChE, T30, or T14 where the C-terminal lysine is either absent or capped by tryptophan [7]. Moreover, immuno-neutralising the antibody by prior complexing with exogenous T14 abolishes all staining in immunohistochemistry, confirming that the antibody is specific to the T14 sequence [8].

### 3.2. The mTORC1 Pathway and the T14 Signalling System

**Figure A3:**
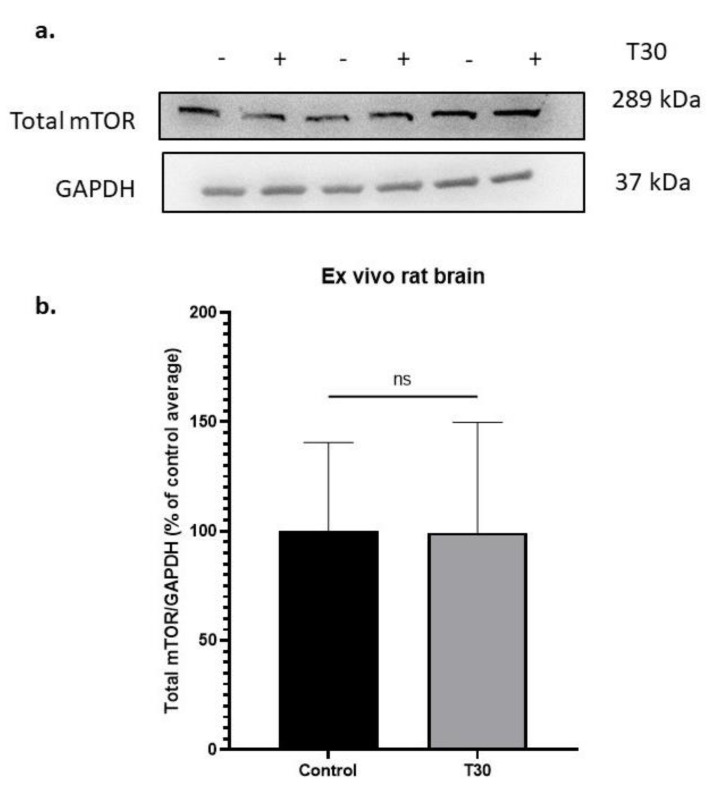
T30 does not affect total mTOR in rat brain ex vivo. (**a**) Representative Western blot image showing mTOR (289 kDa) and GAPDH (37 kDa) for T30 (2 uM) vs. control treated rat brain slices containing substantia nigra. (**b**) Average protein expression of mTOR in control vs. T30 treated rat brain slices containing substantia nigra. The error bars in each case represent the standard deviation (SD). *n* = 6. ns: non-significant based on *t*-test.

The mTORC1 pathway is suggested to be driving both AD and cancer [22,24], and it is possible that its hyperactivation in both pathologies results from the same, albeit yet unidentified, trigger [23]. Proteomic analysis of T30 application in SH-SY5Y cells has shown that the mTOR pathway is involved in the downstream effect of T30 application, and T30 mediates neurite growth through autophagosome and protein synthesis [2,3,26]. As seen here in differentiated PC12 cells, as well as in substantia nigra of rat brain ex vivo, T30 induced an increase in p-mTOR s2448 upon 5–6-h application but did not change total mTOR (Figure A3). Although it is possible that this effect ultimately triggers different cascades, leading to the different pathologies [8], mTORC1 hyperactivation is nonetheless the first pathway identified which links the involvement of T14 induced developmental mechanism in both AD and cancer. Since both T14 and p-mTOR are correlated in the midbrain of AD patients, this further suggests that T14 could be the ultimate trigger for mTORC1 activation in patients with AD. These findings are supported yet again by the increase in phosphorylation of translation regulation factor elF4E (p-elF4E) [26], accompanied by a decrease in autophagy marker-cytosolic light chain (LC3-B-II) reported upon T30 application in the human neural cell line—SH-SY5Y [26]. Both these processes, an increase in p-elF4E and a decrease in LC3-B-II are known to be regulated downstream of mTORC1 activation [26,49,50,51]. It is proposed that during development, T14 may act via α-7 nAChR to activate the mTORC1 to drive neural growth [2,3,48], however during late adulthood, this trophic process turns toxic [1,2,4]. The T14-induced calcium influx is excitotoxic when activated in the context of a mature system [4], which leads to hyperactivation of the mTORC1, culminating in the pathology of Alzheimer’s disease.

**Figure 6:**
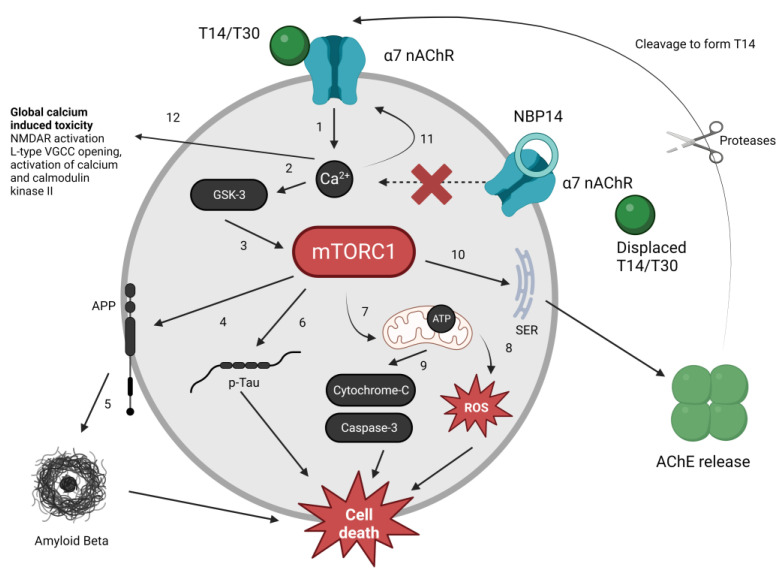
Proposed role of mTORC1 in modulating different intracellular cascades activated by T14. (1) T14 binds to the α-7 nAChR, inducing excess calcium influx [7]. (2) Aberrantly raised calcium triggers the activation of GSK-3 [7], (3) which is known to have an amplificatory effect on the activation of mTORC1 [57]. (4) mTORC1 leads to the production of the amyloid precursor protein (APP), (5) which leads to an increased production of amyloid beta [58]. Both mTORC1 and T14 application have been independently shown to increase the expression of APP and amyloid beta [7,58]. (6) mTORC1 is also known to drive an increase in phosphorylated tau (p-Tau) [59], which is also known to be increased by T14 [7]. (7) mTORC1 also affects mitochondrial dysfunction [17], (8) leading to the generation of ROS. T14-induced calcium influx is known to affect mitochondria and decrease ATP synthesis, causing electron leakage, (9) triggering cytochrome C release, followed by caspase 3 activation, and increasing free radicals [2,8]. Ultimately, the increased expression of amyloid beta, p-Tau, free radicals, and caspase-3 is toxic to the cells, leading to cell death [8]. (10) mTORC1 activation triggers AChE release [9] from intracellular storage e.g., the dendritic smooth endoplasmic reticulum into extracellular space; subsequently proteases e.g., IDE cleave T14 from AChE [60]. T14 diffuses into extra-synaptic space to act on α-7 nAChR, perpetuating the cycle in neighbouring cells. (11) NBP14 displaces T14/T30 on the α-7 nAChR [11], thus blocking the influx of calcium into cells, and ultimately blocking all downstream intracellular cascades known to be activated by T14. (12) T14 induced calcium influx has global toxic effects on cell physiology, such as NMDAR activation, opening of VGCC, and generation of calcium and calmodulin kinase II [4].

The next crucial question is whether mTOR activation is an essential component of the T14/T30-induced intracellular mechanism. Rapamycin blocked the effects of T30 in PC12 cells, as measured by AChE release, thus suggesting that the activation of the mTOR pathway is indeed important for T30 to mediate its downstream effects. In cells with reduced *Rictor* expression, essential for mTORC2 formation [37], the release of AChE in PC12 cells following T30 administration was unaffected. On the other hand, silencing *Raptor*, required for mTORC1 formation [37], reduced the release of AChE following T30 administration compared to control cells. Silencing *Raptor* had no effects on mTORC2 whilst silencing *Rictor* had no effects on mTORC1 (see Figure A1 and Figure A2). These data suggest that T30 requires the independent activation of the mTORC1 pathway, but not the mTORC2 pathway, to mediate its downstream effects on AChE release. Figure 6 summarises the role of mTORC1 in different intracellular cascades previously known to be activated by T14, and the proposed interaction of these with T14-induced processes.

Rapamycin has been suggested as a potential therapy for AD [52], and other blockers of the mTOR pathway, such as everolimus, are already approved therapies for certain types of cancer [53]. However, these drugs target both mTORC1 and mTORC2 [27,30,31,33], and pre-clinical and clinical studies have shown that the complete blockade of the mTOR pathway may not be the most efficient therapy for these conditions [32]. Firstly, the complete blockade of the mTOR pathway is detrimental, since basal levels of mTOR are required for normal function of cells [17]. Secondly, and most importantly, drugs such as rapamycin are not specific, with off-target effects related to the mTORC2 [36], thus leading to various severe side-effects such as diabetes, and anaemia [31], which are linked to rapamycin’s ability to block cell proliferation [31,44]. Thus, interception of the primary, extracellular trigger of the mTORC1 hyperactivation would be a better alternative therapy for these pathologies. Here, we propose that using NBP14 or comparable T14 blockade could yield improved results in treating these therapies compared to established mTOR blockers. Moreover, given the effect of rapamycin on cell growth [44], its off-target effects on mTORC2 [36], and given the requirement for basal activity of the mTOR pathway for cell survival and function [19], it could be that blockade of T14 does not have comparable side effects. Thus, there is a possibility that T14 blockade might be a preferable alternative for treating a range of diverse conditions and applications which respond to rapamycin, such as coating of stents [54], immunosuppression [55], and anti-ageing [56] among other wider applications. We have shown that NBP14 alone has no effect in PC12 cells across a range of parameters [9], and in ex vivo brain slices when applied alone [10]. The drug is an inert antagonist as it only acts to block the effects of its linear counterpart. As seen here, rapamycin reduces the proliferation of PC12 cells, whereas NBP14 does not. Therefore, use of NBP14 may have an improved therapeutic potential in blocking the aberrant activation of the mTORC1 induced by T14, without the side effects such as diabetes, anaemia, thrombocytopaenia, among other severe side effects associated with rapamycin.

## 4. Materials and Methods

### 4.1. PC12 Cell Culture and Reagents

PC12 cells are an immortalised pheochromocytoma cell line derived from the rat adrenal medulla [42]. They display several properties that make them suitable for studying neuronal function as they can be differentiated by nerve growth factor (NGF) treatment to display neuronal behaviour [39]. Wild-type PC12 cells were purchased from Sigma (Merck, kGaA, Darmstadt, Germany, 88022401). The culture was routinely plated on 100 mm dishes (Corning, Somerville, MA, USA, StarLab CC7682-3394) coated with type IV collagen from human placenta (2 mg/cm^2^, Sigma, Merck, kGaA, Darmstadt, Germany, C5533) and maintained in full growth medium with Dulbecco’s Modified Eagle Medium (DMEM)—high glucose (Sigma, Merch, kGaA, Darmstadt, Germany, 11574456) supplemented with 10% heat-inactivated horse serum (HS) and 5% Foetal Bovine Serum (FBS), 0.1 mg/mL penicillin/streptomycin, and 2.5 ug/mL amphotericin B. The medium was changed every 2–3 days and cells were maintained in a humidified incubator at 37 °C with 5% CO_2_. For passaging, the cells were scraped from a dish using a cell scraper and passed through a syringe and needle to obtain a single cell suspension. For differentiation, the cells were cultured in differentiation media containing high glucose DMEM, 1% heat-inactivated HS, 0.5% FBS, 0.1 mg/mL penicillin/streptomycin and 2.5 ug/mL amphotericin B with 200 ng/mL 2.5S NGF from murine submaxillary gland (Sigma, Merck, kGaA, Darmstadt, Germany). The passage number of cells used for experiments varied between 12–20.

### 4.2. Ex Vivo Brain Slices

**Figure 7:**
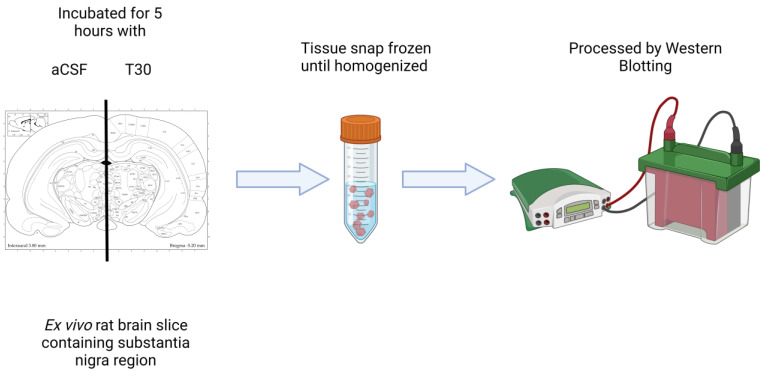
Summary of the experimental method used for ex-vivo brain slice incubation.

Postnatal day 21 (P21) male Wistar rats were used for these experiments (Charles River, Harlow, UK). The procedures of animal experimentation were approved and performed in accordance with the UK Home Office regulations (“Schedule 1”) and conducted in compliance with the requirements of the UK Animals (Scientific Procedures) Act 1986. The brain dissection, slicing and incubation was performed as previously described [11]. Briefly, a measured quantity of isoflurane was administered (100% *w*/*w*) to induce anaesthesia and absence of pedal withdrawal reflex established its correct levels. According to the guidance provided by Schedule 1, cervical dislocation was then performed prior to brain extraction. The brain was removed and kept in ice-cold slicing artificial cerebrospinal fluid (aCSF). Subsequently, the brain was vibratome-sliced with a Leica VT1000S vibrating microtome and three consecutive sections (400 µm thick) were collected within the following stereotaxic coordinates: −4.80 to −6.20 mm from Bregma [61] containing substantia nigra (SN). Afterwards, each slice was divided along the midline to provide two complementary halves of the same anatomical plane. Successively, three serial hemi-sections were incubated, for 5 h. Depending on the treatment, the brain tissue was incubated with “recording” aCSF alone or treated with T30. The working concentrations (mmol) of the two aCSFs, previously described [11], are the following: slicing aCSF: 120 NaCl, 5 KCl, 20 NaHCO_3_, 2.4 CaCl_2_, 2 MgSO_4_, 1.2 KH_2_PO_4_ and 10 glucose; 6.7 HEPES salt and 3.3 HEPES acid; pH: 7.1. Recording aCSF: 124 NaCl, 3.7 KCl, 26 NaHCO_3_, 2 CaCl_2_, 1.3 MgSO_4_, 1.3 KH_2_PO_4_, and 10 glucose; pH: 7.1. The aCSF solution was at all times bubbled with a mixture of 95% O_2_ with 5% CO_2_ to ensure appropriate levels of oxygenation. Following the incubation with either aCSF or T30, the brain slices were snap frozen for tissue homogenization and protein quantification (Figure 7).

### 4.3. Cell Viability Assay

The number of viable cells was determined using the cell counting kit-8 (CCK-8; Sigma-Aldrich, Merck, kGaA, Darmstadt, Germany, 96992). PC12 cells were plated in a 96-well plate with 1 µM rapamycin (Bio-Techne, Minneapolis, MN, USA, Tocris, 1292, dissolved in DMSO) or vehicle (with 0.0005% *v*/*v* DMSO) or 1 µM NBP14 (dissolved in water) or vehicle conditions in full culture media. CCK-8 reagent was added to the wells (10% *v*/*v*) 24 or 48 h after plating. The plate was then incubated at 37 °C for 1 h before the absorbance was detected at 450 nm in a CLARIOstar Plus plate reader (BMG Labtech, Aylesbury, UK). For data analysis, each value was represented as a percentage of control to account for inter-assay variability.

### 4.4. AChE Release Assay

MAK119 AChE assay kit from Sigma-Aldrich (St. Louis, MO, USA), which is based on the Ellman assay, was used to determine the AChE release by the cells. Thiocholine, produced by AChE, reacts with 2-nitrobenzoic acid, which forms a yellow-coloured product directly proportional to the activity of enzyme AChE. PC12 cells were plated in the middle 60 wells of a 96 well microplate in 100 uL of differentiation media 5 days before the experiment was conducted. Twenty-four hours before the experiment, rapamycin (1 µM, dissolved in 0.0005% DMSO *v*/*v*) or vehicle control (0.0005% DMSO in full cell culture media) was added to the wells [26]. On the day of the experiment, treatments with control (0.0005% *v*/*v* DMSO and 0.002% *v*/*v* acetonitrile), T30 (40 µM, 0.002% *v*/*v* acetonitrile), rapamycin (1 µM) + T30 (40 µM), rapamycin alone (1 µM), or T30 (40 µM) + NBP14 (10 µM) were prepared in Hanks’ balanced salt solution (HHBS) supplemented with 20 mM HEPES. The media was removed, and the cells were treated with 40 uL of treatment prepared in HHBS per well. After 3.5 h of incubation in the incubator at 37 °C, the supernatant was removed from each well. Supernatants from two wells with the same conditions were combined, which was dispensed into a 96 well microplate. To each well, assay reagent dissolved in assay buffer was added. The plate was then incubated for 2 min at room temperature, following which the absorbance was read at 405 nm in a CLARIOstar Plus plate reader (BMG Labtech, Aylesbury, UK), followed by a final reading 8 min after the first reading. For data-analysis, the activity of AChE was measured by subtracting the initial absorbance from the final absorbance, and each well was represented as a percentage of their respective controls to control for inter-assay variability.

### 4.5. siRNA Transfection 

Cells were plated 18–20 h before transfection in a 96-well plate (6700 cells per well) in full DMEM supplemented with 10% HS, 5% FBS, 0.1 mg/mL penicillin/streptomycin, and 2.5 ug/mL amphotericin B. On the day of transfection, full DMEM was replaced by serum- and antibiotic-free DMEM. *Raptor* siRNA (siRNA ID: s143003) and *Rictor* siRNA (siRNA ID: s160197; Invitrogen, Thermo Fisher Scientific, Swindon, UK) were transfected using Lipofectamine RNAiMAX transfection reagent according to the manufacturer’s instructions. In each well of a 96-well plate, the cells received a final volume of 0.2 uL of Lipofectamine RNAiMAX and 40 nM of the siRNA diluted in Opti-MEM. The medium was replaced with full serum and antibiotic medium 6 h after the transfection. The cells were used for either the AChE assay, or harvested for protein or RNA extraction 72 h after transfection.

### 4.6. Real-Time Quantitative Polymerase Chain Reaction (RT-qPCR)

Total RNA was extracted using the RNeasy Plus Mini Kit (Qiagen, Manchester, UK) according to manufacturer’s instructions. cDNA synthesis was conducted using the qScript cDNA Synthesis Kit (95047, QuantaBio, Beverly, MA, USA) according to the manufacturer’s protocol, using 300 ng RNA. RT-qPCR was conducted on a Q thermal cycler (Quantabio, Beverly, MA, USA) using primers listed in Table 1 and the PerfeCTa SYBR Green FastMix (Quantabio, #95074-012, Beverly, MA, USA), according to the manufacturer’s protocol. Relative mRNA expression was quantified using the Q software (v1.0.4, Quantabio, Beverly, MA, USA) with the standard curve method, and target gene expression was normalised to GAPDH and beta-actin expression.

### 4.7. Human Clinical Samples

Midbrain blocks of fresh-frozen tissue were supplied by the Thomas Willis Oxford Brain Collection (c/o Professor Margaret Esiri). An ethics application was approved by London—City and East NRES Committee on behalf of the Human Tissue Bank of Oxford Radcliffe Hospital NHS that complied with the Human Tissue Act, Human Tissue Authority Codes of Practice and other law relevant to post-mortem examinations and use of tissue. Ethics committee number: 08/H0704/128+5. Approval code: 07/Q1605/16. Approval date: 2 December 2015.

### 4.8. Western Blotting

Human brain tissue, rat brain tissue, and PC12 cells were lysed using RIPA lysis and extraction buffer (Thermo Fisher Scientific, Swindon, UK), supplemented with protease (cOmplete™ ULTRA Tablets, Mini, EDTA-free, EASYpack Protease Inhibitor Cocktail, Roche, Basel, Switzerland) and phosphatase (PhosSTOP, Roche) inhibitor cocktails. Human brain was homogenized by mechanical disruption using a KIMBLE Dounce tissue grinder set (Sigma, Merck, kGaA, Darmstadt, Germany, D9063) and centrifuged at 13,000 RPM at 4 °C for 40 min. Rat brain tissue and PC12 cell lysates were disrupted using pestles, and centrifuged at 13,000 RPM at 4 °C for 10 min. The supernatant was collected, and protein was quantified using the Thermo Scientific Pierce 660 nm Protein Assay. Equal amounts of protein (50 µg for human brain, 30 µg for rat brain, and 12 µg for PC12 cell lysates) were mixed with 4X Laemmli sample buffer (BioRad, Watford, UK, 1610747) and 10% (*v*/*v*) of 2-mercaptoethanol. The protein sample was then heated at 60 °C for 10 min and loaded onto a 4 to 20% Mini-PROTEAN TGX Precast Gel (15 well or 10 well gel) (BioRad, UK, 4561093). Proteins were separated by electrophoresis and transferred from gels to polyvinylidene fluoride (PVDF) membranes. The No-Stain™ Protein Labelling Reagent (ThermoFisher Scientific, Waltham, MA, USA, A44717) was used to stain the total protein transferred to the membrane. Briefly, after the transfer, the membrane was washed with water and stained with the labelling reagent for 10 min. The membrane was washed with water again, and total protein content was visualized using a CCD Camera using an epi long wave UV filter (G-Box, Syngene, Cambridge, UK). The membrane was then blocked at room temperature for 1 h in 5% defatted milk in Tris-buffered saline plus 0.05% Tween 20 (TBS-T). The membranes were then incubated in primary antibody, diluted in blocking solution overnight at 4 °C with gentle agitation. The primary antibodies used were against T14 (1:1000, custom made by Genosphere), p-mTOR s2448 (1:2000, Abcam, Cambridge, UK, ab109268), p-mTOR s2481 (1:2000, Abcam, ab137133) and GAPDH (1:15,000, Abcam, ab181602). After primary antibody incubation overnight, the membranes were washed three times with TBS-T for 5 min and incubated with secondary goat-anti-rabbit IgG H + L Horseradish peroxidase (HRP) conjugated antibody (1:10,000, Thermo Fisher Scientific, G21234), diluted in blocking solution, for 1 h at room temperature, with gentle agitation. The membranes were then washed three times with TBS-T for 5 min, followed by one wash with TBS for 5 min. After washing, proteins were visualized using an enhanced chemiluminescence-based detection kit following the manufacturer’s protocol (BioRad, UK, 1705061) and a CCD Camera (G-Box, Syngene, Cambridge, UK) gel system. Band intensities were quantified using ImageJ and target proteins were normalised to GAPDH, or total protein levels.

### 4.9. Statistical Analysis

For cell culture assays, the data are represented as a percentage of the control average and the errors represent the standard deviation (SD). Wherever applicable, an unpaired *t*-test or a one sample *t*-test was performed if there were two groups, or a one-way ANOVA was performed if there were more than two groups, followed by a Dunnett’s post-hoc test. To assess the possible correlation between T14 and p-mTOR, the data was log transformed followed by a Pearson’s correlation test (two-tailed). All analysis was conducted in GraphPad PRISM and *p* < 0.05 is considered statistically significant throughout.

## 5. Conclusions

This study has shown that the peptide T14 selectively activates the mTORC1 pathway, and this is essential for its downstream intracellular effects. This observation further confirms the role of T14 as trophic in early development, and its aberrant reactivation as toxic in pathologies such as AD and cancer. We also suggest that blockade of T14, for example by its cyclic variant NBP14, represents a possible therapeutic advance for conditions in which mTORC1 is known to be hyperactivated: displacement of T14 on the α-7 nAChR would more specifically exert its effects on the mTORC1 pathway alone, thereby avoiding any off-target effects on the mTORC2 pathway. T14 blockade could therefore offer a new therapeutic approach for other conditions in which rapamycin, or other general mTOR blockers, have limitations, for example, organ transplant patients, and stent coating, by potentially reducing the side effects associated with generalized mTOR blockade.

## 6. Patents

The work described in this study is covered by international patent application, “WO2015/004430” (Family 1—NBP-14), and GB Patent application, “128708GB1” (Rapamycin sensitive conditions).

## Figures and Tables

**Table 1:** Primers used for RT-qPCR.

Gene	Forward Primer Sequence (5′-3′)	Reverse Primer Sequence (5′-3′)	Product Size (bp)
Raptor	ACGGTGAATGGAGAGGTCTG	CAGCATTGGAGCAGTCGTAG	124
Rictor	TGCTTCCTTGTTTCCGAGTT	GCTACCACCTCTGGGTTCTG	138
GAPDH	GGGCTCTCTGCTCCTCCCTGT	CAGGCGTCCGATACGGCCAAA	119
Beta-actin	CCACACCCGCCACCAGTTCG	TACAGCCCGGGGAGCATCGT	112

## Data Availability

All the data supporting the findings of this study are available within the paper.

## References

[1] Greenfield S., Vaux D.J. (2002). Parkinson’s disease, Alzheimer’s disease and motor neurone disease: Identifying a common mechanism. Neuroscience.

[2] Day T., Greenfield S.A. (2004). Bioactivity of a peptide derived from acetylcholinesterase in hippocampal organotypic cultures. Exp. Brain Res..

[3] Greenfield S.A., Day T., Mann E.O., Bermudez I. (2004). A novel peptide modulates alpha7 nicotinic receptor responses: Implications for a possible trophic-toxic mechanism within the brain. J. Neurochem..

[4] Day T., Greenfield S.A. (2003). A peptide derived from acetylcholinesterase induces neuronal cell death: Characterisation of possible mechanisms. Exp. Brain Res..

[5] Bond C.E., Zimmermann M., Greenfield S.A. (2009). Upregulation of α7 Nicotinic Receptors by Acetylcholinesterase C-Terminal Peptides. PLoS ONE.

[6] Garcia-Ratés S., Lewis M., Worrall R., Greenfield S. (2013). Additive Toxicity of β-Amyloid by a Novel Bioactive Peptide In Vitro: Possible Implications for Alzheimer’s Disease. PLoS ONE.

[7] Garcia-Ratés S., Morrill P., Tu H., Pottiez G., Badin A.-S., Tormo-Garcia C., Heffner C., Coen C.W., Greenfield S.A. (2016). (I) Pharmacological profiling of a novel modulator of the α7 nicotinic receptor: Blockade of a toxic acetylcholinesterase-derived peptide increased in Alzheimer brains. Neuropharmacology.

[8] Greenfield S.A., Cole G.M., Coen C.W., Frautschy S., Singh R.P., Mekkittikul M., Garcia-Ratés S., Morrill P., Hollings O., Passmore M. (2022). A novel process driving Alzheimer’s disease validated in a mouse model: Therapeutic potential. Alzheimer’s Dement. Transl. Res. Clin. Interv..

[9] Hasan S., Ahmed M., Garcia-Ratés S., Greenfield S. (2023). Antagonising a novel toxin “T14” in Alzheimer’s disease: Comparison of receptor blocker versus antibody effects in vitro. Biomed. Pharmacother..

[10] Greenfield S.A., Ferrati G., Coen C.W., Vadisiute A., Molnár Z., Garcia-Rates S., Frautschy S., Cole G.M. (2022). Characterization of a Bioactive Peptide T14 in the Human and Rodent Substantia Nigra: Implications for Neurodegenerative Disease. Int. J. Mol. Sci..

[11] Brai E., Simon F., Cogoni A., Greenfield S.A. (2018). Modulatory Effects of a Novel Cyclized Peptide in Reducing the Expression of Markers Linked to Alzheimer’s Disease. Front. Neurosci..

[12] Onganer P.U., Djamgoz M.B.A., Whyte K., Greenfield S.A. (2006). An acetylcholinesterase-derived peptide inhibits endocytic membrane activity in a human metastatic breast cancer cell line. Biochim. Biophys. Acta BBA—Gen. Subj..

[13] Pepper C., Tu H., Morrill P., Garcia-Rates S., Fegan C., Greenfield S. (2017). Tumor cell migration is inhibited by a novel therapeutic strategy antagonizing the alpha-7 receptor. Oncotarget.

[14] Garcia-Ratés S., Greenfield S. (2017). Cancer and neurodegeneration: Two sides, same coin?. Oncotarget.

[15] Tan V.P., Miyamoto S. (2016). Nutrient-sensing mTORC1, Integration of metabolic and autophagic signals. J. Mol. Cell. Cardiol..

[16] Tokunaga C., Yoshino K., Yonezawa K. (2004). mTOR integrates amino acid- and energy-sensing pathways. Biochem. Biophys. Res. Commun..

[17] Ben-Sahra I., Manning B.D. (2017). mTORC1 signaling and the metabolic control of cell growth. Curr. Opin. Cell. Biol..

[18] Wang X., Proud C.G. (2006). The mTOR Pathway in the Control of Protein Synthesis. Physiology.

[19] Saxton R.A., Sabatini D.M. (2017). mTOR Signaling in Growth, Metabolism, and Disease. Cell.

[20] Yuan H.-X., Guan K.-L. (2016). Structural insights of mTOR complex 1. Cell Res..

[21] Szwed A., Kim E., Jacinto E. (2021). Regulation and metabolic functions of mTORC1 and mTORC2. Physiol. Rev..

[22] Tramutola A., Triplett J.C., Di Domenico F., Niedowicz D.M., Murphy M.P., Coccia R., Perluigi M., Butterfield D.A. (2015). Alteration of mTOR signaling occurs early in the progression of Alzheimer disease (AD): Analysis of brain from subjects with pre-clinical AD, amnestic mild cognitive impairment and late-stage AD. J. Neurochem..

[23] Majd S., Power J., Majd Z. (2019). Alzheimer’s Disease and Cancer: When Two Monsters Cannot Be Together. Front. Neurosci..

[24] De la Cruz López K.G., Toledo Guzmán M.E., Sánchez E.O., García Carrancá A. (2019). mTORC1 as a Regulator of Mitochondrial Functions and a Therapeutic Target in Cancer. Front. Oncol..

[25] Sun Y.-X., Ji X., Mao X., Xie L., Jia J., Galvan V., Greenberg D.A., Jin K. (2013). Differential Activation of mTOR Complex 1 Signaling in Human Brain with Mild to Severe Alzheimer’s Disease. J. Alzheimers Dis..

[26] Graur A., Kabbani N. (2023). The Human Acetylcholinesterase c-Terminal T30 Peptide Activates Neural Growth through an Alpha 7 Nicotinic Acetylcholine Receptor mTOR Pathway. bioRxiv.

[27] Li J., Kim S.G., Blenis J. (2014). Rapamycin: One Drug, Many Effects. Cell Metab..

[28] Fletcher L., Evans T.M., Watts L.T., Jimenez D.F., Digicaylioglu M. (2013). Rapamycin Treatment Improves Neuron Viability in an In Vitro Model of Stroke. PLoS ONE.

[29] Copp J., Manning G., Hunter T. (2009). TORC-Specific Phosphorylation of Mammalian Target of Rapamycin (mTOR): Phospho-Ser2481 Is a Marker for Intact mTOR Signaling Complex 2. Cancer Res..

[30] Verhave J., Boucher A., Dandavino R., Collette S., Senécal L., Hebert M.-J., Girardin C., Cardinal H. (2014). The incidence, management, and evolution of rapamycin-related side effects in kidney transplant recipients. Clin. Transplant..

[31] Soefje S.A., Karnad A., Brenner A.J. (2011). Common toxicities of mammalian target of rapamycin inhibitors. Target. Oncol..

[32] Carosi J.M., Sargeant T.J. (2019). Rapamycin and Alzheimer disease: A double-edged sword?. Autophagy.

[33] Dumas S.N., Lamming D.W. (2020). Next Generation Strategies for Geroprotection via mTORC1 Inhibition. J. Gerontol. Ser. A.

[34] Adebayo Michael A.O., Ko S., Tao J., Moghe A., Yang H., Xu M., Russell J.O., Pradhan-Sundd T., Liu S., Singh S. (2019). Inhibiting Glutamine-Dependent mTORC1 Activation Ameliorates Liver Cancers Driven by β-Catenin Mutations. Cell Metab..

[35] Rosner M., Siegel N., Valli A., Fuchs C., Hengstschläger M. (2010). mTOR phosphorylated at S2448 binds to raptor and rictor. Amino Acids.

[36] Sarbassov D.D., Ali S.M., Sengupta S., Sheen J.H., Hsu P.P., Bagley A.F., Markhard A.L., Sabatini D.M. (2006). Prolonged Rapamycin Treatment Inhibits mTORC2 Assembly, A.k.t./.P.K.B. Mol. Cell.

[37] Sarbassov D.D., Ali S.M., Kim D.H., Guertin D.A., Latek R.R., Erdjument-Bromage H., Tempst P., Sabatini D.M. (2004). Rictor, a Novel Binding Partner of mTOR, Defines a Rapamycin-Insensitive and Raptor-Independent Pathway that Regulates the Cytoskeleton. Curr. Biol..

[38] Westerink R.H.S., Ewing A.G. (2007). The PC12 cell as model for neurosecretion. Acta Physiol..

[39] Teng K., Angelastro J., Cunningham M., Greene L. (2006). Cultured PC12 Cells A Model for Neuronal Function, Differentiation, and Survival. Cell Biology.

[40] Youdim M.B.H. (1991). PC12 cells as a window for the differentiation of neural crest into adrenergic nerve ending and adrenal medulla. Recent Advances in Neuropharmacology.

[41] Melega W.P., Howard B.D. (1981). Choline and acetylcholine metabolism in PC12 secretory cells. Biochemistry.

[42] Wiatrak B., Kubis-Kubiak A., Piwowar A., Barg E. (2020). PC12 Cell Line: Cell Types, Coating of Culture Vessels, Differentiation and Other Culture Conditions. Cells.

[43] Izumiyama N., Asami E., Itoh Y., Ohtsubo K. (1990). Alzheimer’s neurofibrillary tangles and paired helical filaments in the pheochromocytoma cells of the adrenal medulla ?Electron microscopic and immunoelectron microscopic observations. Acta Neuropathol..

[44] Parker E.M., Monopoli A., Ongini E., Lozza G., Babij C.M. (2000). Rapamycin, but not FK506 and GPI-1046, increases neurite outgrowth in PC12 cells by inhibiting cell cycle progression. Neuropharmacology.

[45] Ferrati G., Brai E., Stuart S., Marino C., Greenfield S. (2018). A Multidisciplinary Approach Reveals an Age-Dependent Expression of a Novel Bioactive Peptide, Already Involved in Neurodegeneration, in the Postnatal Rat Forebrain. Brain Sci..

[46] Uematsu M., Nakamura A., Ebashi M., Hirokawa K., Takahashi R., Uchihara T. (2018). Brainstem tau pathology in Alzheimer’s disease is characterized by increase of three repeat tau and independent of amyloid β. Acta Neuropathol. Commun..

[47] Woolf N.J. (1996). Global and serial neurons form a hierarchically arranged interface proposed to underlie memory and cognition. Neuroscience.

[48] Garcia-Ratés S., Greenfield S. (2022). When a trophic process turns toxic: Alzheimer’s disease as an aberrant recapitulation of a developmental mechanism. Int. J. Biochem. Cell Biol..

[49] Kosciuczuk E.M., Saleiro D., Platanias L.C. (2017). Dual targeting of eIF4E by blocking MNK and mTOR pathways in leukemia. Cytokine.

[50] Majeed S.T., Batool A., Majeed R., Bhat N.N., Zargar M.A., Andrabi K.I. (2021). mTORC1 induces eukaryotic translation initiation factor 4E interaction with TOS-S6 kinase 1 and its activation. Cell Cycle.

[51] Deleyto-Seldas N., Efeyan A. (2021). The mTOR–Autophagy Axis and the Control of Metabolism. Front. Cell Dev. Biol..

[52] Kaeberlein M., Galvan V. (2019). Rapamycin and Alzheimer’s disease: Time for a clinical trial?. Sci. Transl. Med..

[53] Paplomata E., Zelnak A., O’Regan R. (2013). Everolimus: Side effect profile and management of toxicities in breast cancer. Breast Cancer Res. Treat..

[54] Reho J.J., Guo D.-F., Morgan D.A., Rahmouni K. (2021). mTORC1 (Mechanistic Target of Rapamycin Complex 1) Signaling in Endothelial and Smooth Muscle Cells Is Required for Vascular Function. Hypertension.

[55] Hahn D., Hodson E.M., Hamiwka L.A., Lee V.W., Chapman J.R., Craig J.C., Webster A.C. (2019). Target of rapamycin inhibitors (TOR-I; sirolimus and everolimus) for primary immunosuppression in kidney transplant recipients. Cochrane Database Syst. Rev..

[56] Selvarani R., Mohammed S., Richardson A. (2021). Effect of rapamycin on aging and age-related diseases—Past and future. Geroscience.

[57] Evangelisti C., Chiarini F., Paganelli F., Marmiroli S., Martelli A.M. (2020). Crosstalks of GSK3 signaling with the mTOR network and effects on targeted therapy of cancer. Biochim. Biophys. Acta BBA—Mol. Cell Res..

[58] Cai Z., Zhou Y., Xiao M., Yan L.-J., He W. (2015). Activation of mTOR: A culprit of Alzheimer’s disease?. Neuropsychiatr. Dis. Treat..

[59] Tang Z., Bereczki E., Zhang H., Wang S., Li C., Ji X., Branca R., Lehtiö J., Guan Z., Filipcik P. (2013). Mammalian Target of Rapamycin (mTor) Mediates Tau Protein Dyshomeostasis. J. Biol. Chem..

[60] Jean L., Thomas B., Tahiri-Alaoui A., Shaw M., Vaux D.J. (2007). Heterologous Amyloid Seeding: Revisiting the Role of Acetylcholinesterase in Alzheimer’s Disease. PLoS ONE.

[61] Paxinos G., Watson C. (2006). The Rat Brain in Stereotaxic Coordinates.

